# Institutional Notes and News

**Published:** 1910-08-06

**Authors:** 


### Institutional Notes and News.

**Hospital Extension Scheme for Coventry.**

On Tuesday the 26th ult. a town meeting, convened by the Mayor, Alderman Lee, at the request of a representative body of the members of the general committee of the Coventry and Warwickshire Hospital, was held at Coventry to consider a scheme for the extension of the institution, which is inadequate for the demands upon it. There was a very large and thoroughly representative gathering, and considerable interest was manifested in the speeches. Col. Wyley proposed the following resolution: "That in order to perpetuate the memory of our late King Edward VII., during whose reign the city of Coventry largely increased in prosperity and population, a new wing, to be called the King Edward VII. wing, be added to the hospital together with other necessary additions and alterations," and explained that a sum of £25,000 was wanted. Mr. Vernon Pugh seconded, and Sir Henry Burdett supported the resolution, the latter pointing out that the hospital accommodation of the city was utterly inadequate for the hospital population, and that in order to provide for the latter more than a hundred additional beds were required. An attempt was made to move a resolution to the effect that hospitals should be State supported, but this was ruled out of order. Canon Masterman, Dr. Lynes, and other speakers supported the original resolution, which was carried. A preliminary committee was formed, with power to add to its number, for the purpose of collecting funds for the scheme.

**The Leicester Infirmary Reconstruction Scheme.**

With a donation of £105 generously granted by the directors of the Stamford, Spalding, and Boston Bank, Ltd., and £105 promised by Ald. Wherry, J.P., of Bourne, Sir Edward Wood, the Chairman of the Board of Governors of the Leicester Infirmary, was able to announce to the governors that the sum required for the reconstruction scheme (£100,000) had now been promised. There were, however, several structural improvements which might still be effected, but the Board felt it could not undertake their provision for some years to come, and hoped a benefactor would come forward with a proposal to carry out these further requirements. £30,000 could be expended to the utmost advantage of the institution. Efforts must now be directed to increasing the income of the institution; and he estimated that a further £3,000 per annum was necessary to maintain the institution in an efficient manner.

**Leicester Framework Knitters' Homes.**

The new "Corah" hall in connection with these Homes, which had been so generously erected by Messrs. J. A. Corah and Alfred Corah, Past Masters of the Worshipful Company of Framework Knitters, was opened on Monday, July 25 last. Mr. Alexander Lorrimer, the Worshipful Master of the Company, presided, and was supported by a large number of assembled guests. The Chairman, in proposing thanks to "The Donors of the Corah Hall," alluded to the great part played by Messrs. Corah in the industrial life of the town of Leicester, and to the efforts they had displayed in connection with the transference of the Company's Homes to the central city of the industry. The hall which they had so generously provided would be used for services, entertainments, etc., and would largely assist in making the lives of the pensioners peaceful and bright. The kind thoughtfulness of Messrs. Corah was but another instance of their large-heartedness and their generosity.

**Doncaster Royal Infirmary.**

The Doncaster Royal Infirmary is confronted with the problem of extending its buildings, to meet the demand on its present insufficient accommodation, or of building a new infirmary on a new site. The committee which has been sitting to decide the merits of the two schemes is in favour of the latter, because the site on which the present infirmary stands does not lend itself to extension. At present there are only forty-two beds, and the development of the collieries in the district has been one cause of the increased number of cases treated. It has been suggested by one of the hon. medical staff of the infirmary that the pressure on its accommodation could be relieved if the new colliery districts followed the example of Conisborough and built cottage hospitals to receive the less serious cases. Such cottage hospital, or hospitals, could be supported by a subscription of a halfpenny a week from the miners as the Fullerton Hospital at Conisborough is supported now. At the moment some £700 is in hand towards the cost of a new infirmary, which, on the plan of one bed per thousand of the population, should contain 100, or at least 70 beds. The matter is not less urgent because five years ago an enlargement of the present infirmary took place. The fact that the growth of the mining population has made that enlargement insufficient, on a site that has never been very readily adaptable, should make both miners and employers recognise the necessity for coming to the rescue now. Are there indeed two schemes, when one has been tried already without the problem having been solved?

**Birmingham and Midland Eye Hospital.**

Just a year ago the nurses and servants of the Birmingham and Midland Eye Hospital moved into fresh quarters in the new wing. A year's experience has shown how advantageous the move has proved to all parties. The four private wards which were opened last November have proved also a great success. Since there is often some discussion as to what fee should be charged in the pay wards, we quote the following from the annual report: "As was anticipated, the institution of these wards is proving of great benefit to those who are too well off to be hospital patients, and not in a position to have operations at their homes or in private nursing establishments. The fee charged is two guineas per week." It certainly seems that a higher fee would bring the pay wards into competition with nursing homes, and also would exclude a class of paying patient who deserves consideration, and for whom surely the pay ward exists. The quotation just given hits off this class admirably and supports the contention made by Dr. Rees, of the Albert Edward Infirmary, Wigan, to be found in this column of our issue of July 9. The operating theatre has been modernised at a cost of £1,000. The number of in-patients was 1,296, including 19 private patients, while 30,771 out-patients were received. Lord Cobham, who has just ended his year of presidency, praised the beds for paying patients, and suggested that either the ratepayers or the Exchequer should pay for the children's spectacles which were provided as the result of medical inspection. The ordinary income for the year was £6,990, and the ordinary expenditure £8,102.

